# Exploring Health Literacy Among Parents of Children Who Attended the Pediatric Rehabilitation Clinics in Qatar: A Convergent Parallel Research Design

**DOI:** 10.1177/23779608251362293

**Published:** 2025-07-23

**Authors:** Jessie Johnson, Fadi Khraim, Carolyn Wolsey, Rami A. Elshatarat, Lisa Thornton, Dina Schnurman, Mohammed AlDalaykeh, Amal Al-Farsi

**Affiliations:** 1 Faculty of Nursing, Beal University, Sackville, New Brunswick, Canada; 2 College of Nursing, 61780 Qatar University, Doha, Qatar; 3 School of Nursing, 3925 University of Tasmania, Hobart, Tasmania, Australia; 4 College of Nursing, 123305 Taibah University, Madinah, Saudi Arabia; 5 Department of Pediatric Rehabilitation, Sidra Medicine, Doha, Qatar; 6 Department of Pediatrics, Weill Cornell Medical College, Doha, Qatar

**Keywords:** health literacy, pediatric rehabilitation, mixed-method study, communicative health literacy, parental empowerment, critical health literacy, pediatric healthcare

## Abstract

**Background:**

Parents of children treated in rehabilitation settings are required to have heightened health literacy (HL) skills due to the complexity of healthcare provision among this patient population. This novel mixed-method study explores HL among parents of children treated in a pediatric rehabilitation specialty clinic in Qatar, focusing on both Arabic and English-speaking parents. The primary objective is to assess HL among parents of children attending pediatric rehabilitation clinics in Qatar, examining key domains such as functional HL, critical HL, empowerment, and communicative HL.

**Methods:**

Participants were recruited through a self-selected sampling method, with data collected via the All Aspects of Health Literacy scale and qualitative interviews. The study was conducted between November 2021 and May 2022. The study analyzes the congruence between quantitative and qualitative findings to provide a comprehensive view of HL among caregivers.

**Results:**

The study highlighted the diverse demographics, including caregivers’ educational backgrounds, age distribution, income, and children's diagnoses. While the English-speaking sample demonstrates nuanced comprehension and minimal reliance on external resources, the Arabic sample exhibits challenges in functional and critical HL, with some turning to Google for information. Both groups emphasized empowerment and communicative HL. The study underscores the need for tailored interventions, considering the diverse caregiver landscape, to optimize pediatric healthcare outcomes. The outcomes reveal a convergence of quantitative and qualitative data, indicating elevated HL levels among participants. Communicative HL emerges as a strength, while critical HL displays variability, particularly among Arabic-speaking parents.

**Conclusion:**

This research significantly contributes to understanding HL in pediatric rehabilitation, highlighting the need for tailored interventions considering the diverse parents’ landscape.

## Introduction

In the dynamic realm of pediatric healthcare, parents play a pivotal role as primary caregivers, significantly influencing the wellbeing of children, particularly those with complex health conditions requiring specialized care. Health literacy (HL), defined as the capacity to obtain, understand, and apply health information for informed decision making (Parnell et al., 2019), is a critical factor shaping the quality of care and outcomes in pediatric settings (Liu et al., 2020). For parents, HL encompasses the ability to access, comprehend, and utilize health information to make informed choices about their child's health. This ability is especially vital in pediatric rehabilitation, where parents must navigate complex medical information, adhere to treatment plans, and collaborate with multidisciplinary healthcare teams (de Buhr & Tannen, 2020; Hasannejadasl et al., 2022).

### Review of Literature

The concept of HL extends beyond basic literacy and numeracy skills; it includes the ability to navigate healthcare systems, interpret medical information, and actively participate in healthcare decisions (de Buhr & Tannen, 2020; Netemeyer et al., 2020). In pediatric rehabilitation clinics, which serve children with conditions such as cerebral palsy, spinal cord injuries, and other developmental or orthopedic challenges, parental HL becomes paramount. Effective HL empowers parents to communicate with clinicians, understand treatment protocols, and advocate for their child's needs. However, challenges such as the complexity of medical information, linguistic diversity, and the emotional strain of caregiving can hinder parents’ ability to fully engage in their child's care (Liu et al., 2020; Murugesu et al., 2022).

The importance of HL is further underscored by its impact on healthcare outcomes. Low HL is associated with adverse consequences, including increased hospital visits, higher healthcare expenditures, nonadherence to medical guidance, and poorer self-care (Johnson et al., 2024; Parnell et al., 2019). These challenges highlight the need for tailored health education and interventions to enhance parental HL, particularly in specialized healthcare settings like pediatric rehabilitation clinics.

As a rapidly evolving Gulf nation, Qatar hosts a diverse population with varying healthcare needs, reflected in its pediatric rehabilitation landscape. The country's healthcare system serves children from diverse cultural and linguistic backgrounds, making it imperative to understand the HL dynamics of parents in this context. Such understanding is crucial for developing culturally sensitive strategies and interventions that address the unique needs of this population (Johnson et al., 2024). Furthermore, HL aligns with the United Nations Sustainable Development Goal 3 (SDG 3), which emphasizes health and wellbeing (Cruickshank et al., 2023), making it a priority for Qatar as it continues to invest in its healthcare infrastructure.

In addition to the Health Literacy Skills Framework (Squiers et al., 2012), this study recognizes the importance of integrating critical HL and health equity perspectives to fully capture the structural and intersectional barriers caregivers face. Critical HL extends beyond functional skills to include the ability to critically analyze health information and take action to address health determinants (Leach et al., 2023; Sykes et al., 2025). Similarly, a health equity perspective highlights how language barriers intersect to influence access, comprehension, and utilization of healthcare resources (Pandey et al., 2021; Sykes et al., 2025). These complementary frameworks help contextualize disparities in HL and offer deeper insights into parent caregiver needs within Qatar's diverse healthcare landscape.

Despite the global recognition of HL's importance, there is a notable research gap in understanding its nuances within pediatric rehabilitation clinics, particularly in the Middle Eastern context (Johnson et al., 2024; Nair et al., 2022). While existing studies highlight the significance of parental HL, few have explored its specific challenges and implications in pediatric rehabilitation settings. This study aims to address a critical research gap by investigating parental HL within the unique context of pediatric rehabilitation clinics in Qatar.

The study seeks to assess the HL levels of parents in pediatric rehabilitation clinics using a validated HL survey tool. It also aims to explore the qualitative dimensions of parental HL through in-depth interviews, capturing parents’ experiences, challenges, and coping strategies. Additionally, the study examines the concordance or discordance between quantitative HL scores and qualitative narratives, offering a nuanced understanding of parental HL. Finally, it identifies key factors influencing parental HL within the pediatric rehabilitation context in Qatar (Halvorsen et al., 2020). Using convergent parallel mixed-methods approach, this study aims to unravel the complexities of parental HL in pediatric rehabilitation clinics, offering a comprehensive understanding of parental HL, considering cultural, linguistic, and contextual factors specific to the Qatari healthcare setting. It is hoped that the findings from this study will ultimately help in improving the healthcare experience and outcomes for children under specialized care.

## Material and Methods

### General Health Literacy Framework

**Figure 1.**
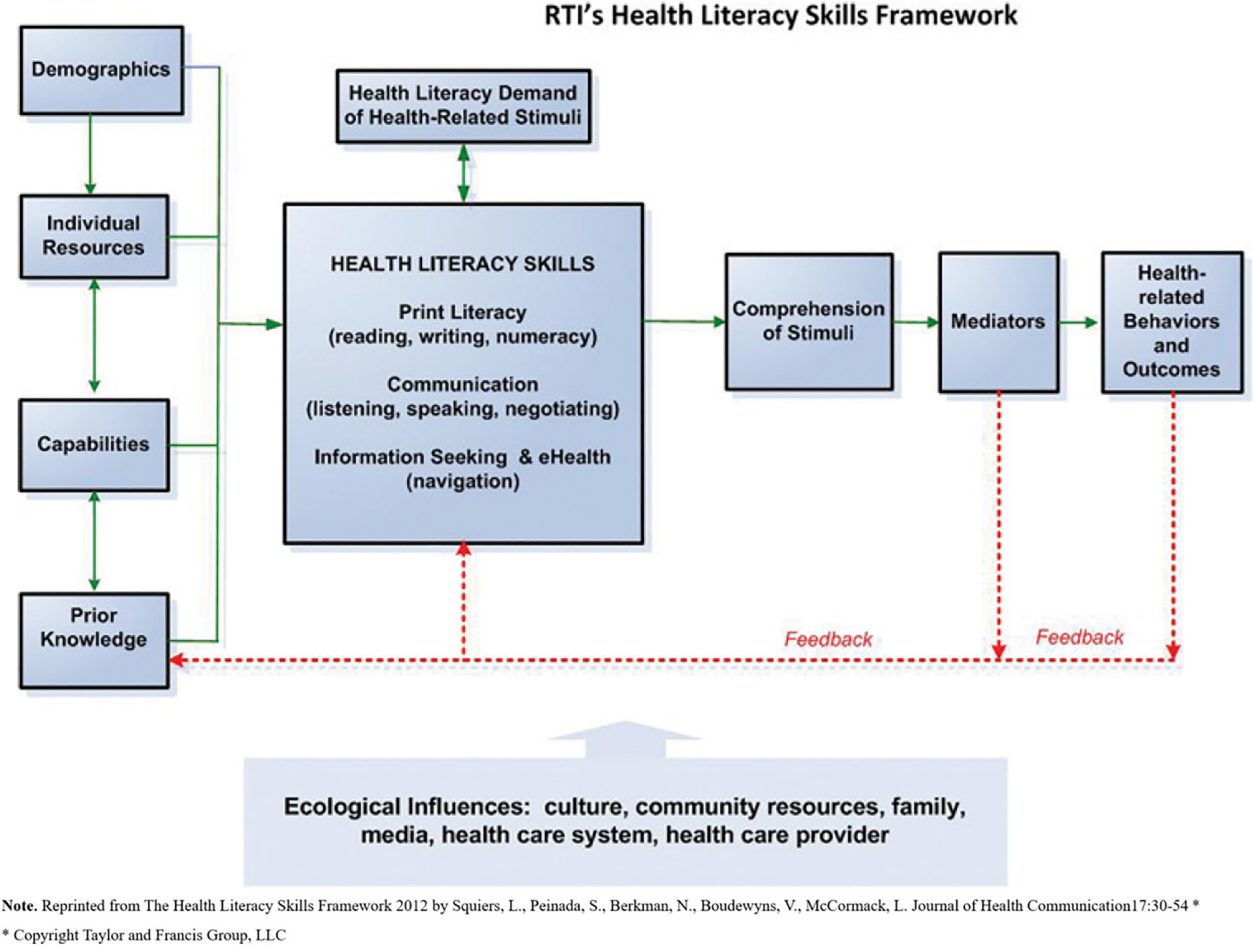
Health

The HL framework employed in this study originated from the Health Literacy Skills conceptual framework (Squiers et al., 2012; Figure 1). This model is a comprehensive understanding of HL development, spanning a continuum. With four core components, the framework highlights the impact of demographics, prior knowledge, resources, and capabilities on the formation and utilization of HL skills. The second component emphasizes essential skills like print literacy, numeracy, and communication in comprehending health-related information. Recognizing the complexity of encountered stimuli, termed HL demand, is the focus of the third component, which interacts with an individual's skills to influence comprehension and decision making (Squiers et al., 2012). The final components delve into comprehension as a primary HL indicator, moderated by stimulus demand, and the subsequent application of these skills in behaviors and outcomes (Squiers et al., 2012). Acknowledging ecological moderators, encompassing cultural, community, family, media, healthcare system, and provider influences, the framework recognizes their role in shaping different aspects of HL. Emphasizing the dynamic nature of these skills, the framework considers their development and deterioration over time based on interactions with health-related stimuli and changing capabilities (Squiers et al., 2012).

Literacy Skills Framework.

### Research Design

**Figure 2.**
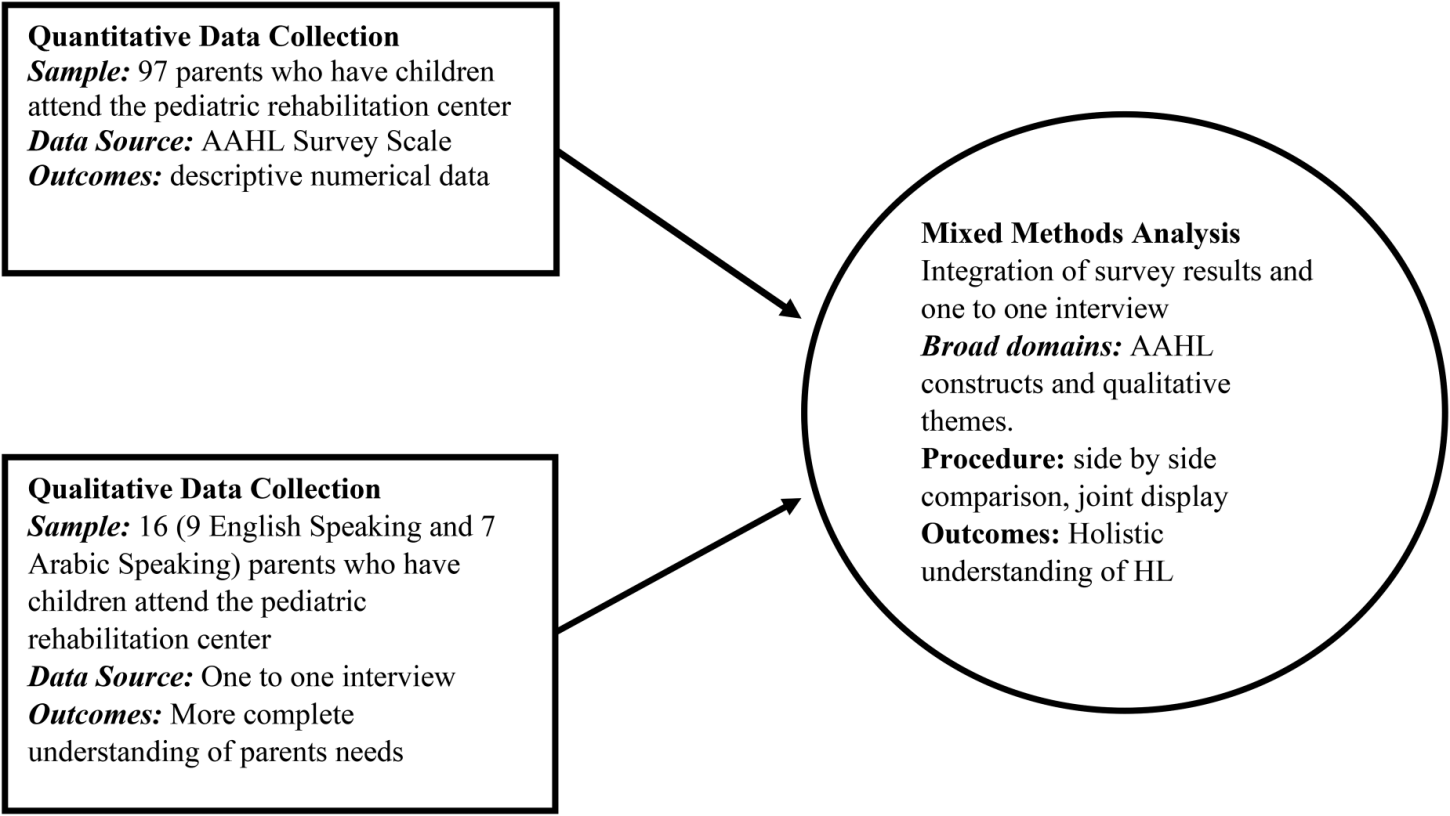
Mixed-Methods Convergent Design.

This study employed a convergent mixed-methods design (Figure 2). Participants included parents of children under 18 attending the Pediatric Rehabilitation Clinic. Participants who completed a quantitative survey featuring the All Aspects of Health Literacy (AAHL) scale (Chinn & McCarthy, 2013), were then invited to participate in in-depth qualitative interviews at a time convenient to them. The interviews aimed to elaborate on their AAHL scale responses and allowed participants to share additional experiences related to HL. The study, involving the surveys and interviews, was conducted between November 2021 and May 2022.

### Research Setting

The study took place at a pediatric rehabilitation clinic in Doha, Qatar, which provides specialized rehabilitation services for children with various health conditions. The clinic, staffed by a single medical specialist, serves children with cerebral palsy and other movement/muscle disorders. Caregivers of children diagnosed with cerebral palsy, genetic disorders (e.g., Phelan McDermid syndrome, congenital disorders of glycosylation), global developmental delay, spinal cord injuries, epilepsy, and other mobility impairments participated. The clinic offered a rich context to explore caregiving experiences and HL factors.

### The Quantitative Survey Study

#### Sample

The study evaluated HL levels among caregivers of children attending a pediatric rehabilitation clinic. All 113 caregivers in the population were invited to participate through online surveys, and 97 of them completed the survey, resulting in a response rate of 86%.

#### Materials

The online survey was administered through QualtricsP^TM^P. Survey links were emailed to caregivers, while clinic receptionists also informed caregivers and provided QR code access to the survey, extending invitations to participate in both the survey and subsequent one-to-one interviews. In the survey, the AAHL scale (Chinn & McCarthy, 2013), a validated 13-item tool, was used to assess HL across four domains:

- Functional literacy (3 items, score range: 3–9): reading and understanding health documents.
- Communicative literacy (3 items, score range: 3–9): communication with health providers.
- Critical literacy (4 items, score range: 3–12): analyzing and managing health information.
- Empowerment literacy (3 items, score range: 3–7): Engaging in community health actions.

The cumulative AAHL scale score, calculated as the sum of scores for each item, served as an indicator for HL, with higher scores reflecting superior HL. The AAHL scale demonstrating adequate reliability with Cronbach alpha value of 0.75 (Chinn & McCarthy, 2013). This study reported a Cronbach alpha of 0.69. In addition to HL assessment, the study gathered sociodemographic data, providing valuable insights into predictors of HL among family caregivers. This encompassed factors such as age, sex, education level, professional preparation (healthcare and nonhealthcare), employment status, household income, number of children, and the age and diagnosis of the child seen at the clinic.

#### Statistical Analysis

IBM SPSS Statistics version 25 was used for quantitative analysis. Descriptive statistics summarized categorical variables (e.g., sex and marital status) as percentages, while continuous variables (e.g., HL scores and age) were reported using mean and standard deviation. This analysis provided a demographic snapshot and HL levels without inferential statistical techniques.

### The Qualitative Interview Study

#### Sample

Convenience sampling was used to recruit caregivers who had completed the quantitative survey and consented to participate in qualitative interviews. Of the 97 participants who completed the survey, 16 parents agreed to take part in the interviews, comprising seven Arabic-speaking (n = 7) and nine English-speaking (n = 9) participants.

#### Data Collection

One-on-one interviews were conducted in person or via Microsoft Teams with one of the parents until data saturation was reached, ensuring a thorough exploration of caregivers’ HL experiences. The flexible format of interviewing allowed participants to engage comfortably. Data saturation was determined when no new themes emerged. Arabic-speaking participants were interviewed by an Arabic-speaking interviewer, while English-speaking interviews were done by an English-speaking interviewer.

#### Data Analysis

The data analysis process followed Gale et al.'s (2013) seven-step framework, ensuring a systematic and thorough approach to understanding the data (Gale et al., 2013). To begin, the research team immersed themselves in the data by reading interview transcripts and listening to recordings, allowing them to become familiar with the caregivers’ narratives. This initial immersion was followed by a detailed line-by-line coding process, where two researchers independently coded the data to capture diverse perspectives and enhance reliability.

The coding process unfolded in three stages: open coding to identify initial themes, axial coding to explore connections between themes, and selective coding to refine and consolidate key themes. Throughout this process, the researchers held regular discussions to compare interpretations and reach consensus, ensuring consistency in theme identification. This collaborative effort led to the development of a working analytical framework, which served as a foundation for organizing the data.

Next, the data were systematically organized. Numerical codes were assigned to facilitate identification, and a framework matrix was constructed to summarize the data by category while preserving the participants’ intended meanings. Finally, the researchers interpreted the data, mapping connections between themes, exploring relationships, and examining potential causality.

### Mixed-Methods Integration and Analysis

Following the qualitative data analysis, the integration of quantitative and qualitative data began by aligning the domains of the AAHL scale with the themes and questions explored in the qualitative interviews. This alignment ensured that both datasets addressed complementary aspects of HL within the pediatric rehabilitation context, providing a cohesive framework for analysis.

The merging of quantitative and qualitative findings was achieved through the creation of a joint display (Table 1), which facilitated meta-inferences by presenting integrated results side by side. This visual representation allowed for a systematic comparison of the two datasets, enabling the research team to identify patterns, corroborate findings, and explore discrepancies. For instance, quantitative results indicating high HL levels in a specific domain were compared and contrasted with qualitative narratives that either supported or challenged these findings.

**Table 1.** Data Integration Matrix.

AAHL scale questions	Qualitative interview questions
**Functional health literacy** 1. How often do you need someone to help you when you are given information to read by your doctor, nurse, or pharmacist? 2. When you need help, can you easily get hold of someone to assist you? 3. Do you need help to fill in official documents?	Do you know the name of your child's diagnosis?
**Critical health literacy** 4. Are you someone who likes to find out lots of different information about your health? 5. How often do you think carefully about whether health information makes sense in your particular situation? 6. How often do you try to work out whether information about your health can be trusted? 7. Are you the sort of person who might question your doctor or nurse's advice based on your own research?	Do you use outside resources like Google to help you understand your child's condition or the recommendations that are made by your doctor or therapist?
**Empowerment** 8. Within the past 12 months have you taken action to do something about a health issue?	Do you understand the best ways to help your child improve?
**Communicative health literacy** 9. When you talk to a doctor or nurse, do you give them all the information they need to help you? 10. When you talk to a doctor or nurse, do you ask the questions you need to ask? 11. When you talk to a doctor or nurse, do you make sure they explain anything that you do not understand?	Is there any part of the instructions that you are unsure of or uncomfortable doing? What do you do if you are unsure or uncomfortable with an instruction or recommendation? Do you feel that you understand your child's diagnosis?

AAHL = All Aspects of Health Literacy Scale.

To address any discordance between the datasets, the team employed a rigorous validation process. Discrepancies were carefully examined by revisiting the raw data, re-analyzing survey responses, and cross-checking interview transcripts. For example, if quantitative results suggested high HL levels in communication skills, but qualitative interviews revealed difficulties in understanding medical jargon, the team explored these inconsistencies through iterative discussions and additional thematic analysis. This process ensured that conclusions drawn from one dataset were validated against the other, enhancing the credibility and reliability of the findings.

The integration process not only broadened the insights gained from each method but also addressed potential inconsistencies, ensuring a comprehensive and robust interpretation of HL. By combining the statistical power of the quantitative data with the depth and nuance of the qualitative narratives, the study provided a holistic understanding of caregivers’ HL experiences. This mixed-methods approach strengthened the validity of the findings and offered actionable insights for improving support for caregivers in pediatric rehabilitation settings.

### Ethical Considerations

Interested parent caregivers provided consent, with written consent for interviews and implied consent for surveys. This ensured voluntary participation, autonomy, and privacy. Confidentiality was maintained through secure physical and electronic data storage, with access restricted to authorized authors. Participants were informed of their right to withdraw at any time without consequences.This study was granted ethical approval by the Institutional Review Board of Sidra Medicine in Doha, Qatar (Approval No. 1760350), as well as by the Conjoint Health Research Ethics Board (CHREB) at the University of Calgary, Canada (Approval No. REB21-0413).

## Results

### Characteristics of Parents and Their Children

The results presented in Table 2 offer a detailed snapshot of the sample characteristics encompassing parents and their children in the study. The primary family caregivers exhibited diverse educational backgrounds, with 38% having education up to high school and 62% holding a college degree or higher. The age distribution of caregivers revealed a substantial representation in the 30 to 39 age range (46%). Monthly household income varied, with 44% reporting incomes below 15,000 Qatari Riyal (4,050 USD), and 17% exceeding 30,000 Qatari Riyal (8,100 USD). Surveys were predominantly completed in Arabic (65%), and 14% of caregivers worked in the healthcare sector. The children, on average, were 7 years old (±6 SD), and families had an average of 3 children (±2 SD). Regarding child diagnoses, 33% had cerebral palsy, 20% had genetic disorders, 9% had global developmental delay, 3% had spinal cord injury, and 35% had other conditions. This comprehensive overview underscores the diversity within the study population, crucial for contextualizing the subsequent findings.

**Table 2.** Characteristics of Parents and Their Children.

Variables	n (%) or (mean ± SD)
**Primary family caregiver's education (N** **=** **89)**	
≤ High school	34 (38%)
≥ College degree or higher	55 (62%)
**Primary family caregiver's age (years) (N** **=** **94)**	
Under 20	6 (6%)
20–29	8 (9%)
30–39	43 (46%)
40–49	27 (29%)
≥ 50	10 (11%)
Above 60	
Monthly household income (Qatari Riyal*) (N **=** **87)**	
< 15,000 (4,050 USD)	38 (44%)
15,000–20,000 (4,050–5,400 USD)	22 (25%)
20,001–30,000 (5,400–8,100 USD)	12 (14%)
> 30,000 (8,100 USD)	15 (17%)
**Surveys completed in Arabic (N** **=** **97)**	63 (65%)
**Family caregiver is working in healthcare (N** **=** **88)**	12 (14%)
**Age of the child (mean** **±** **SD)**	(7 ± 6)
**Number of children in the family (mean** **±** **SD)**	(3 ± 2)
**Class of child diagnosis (N** **=** **92)**	
Cerebral palsy	30 (33%)
Genetic disorders (D2hga type 2, Dyggve Melchior Clausen syndrome, Phelan McDermid syndrome, congenital disorders of glycosylation (CDG))	18 (20%)
Global developmental delay	8 (9%)
Spinal cord injury (Spina bifida)	3 (3%)
Other (club foot, epilepsy, dislocated shoulder, hyper joint mobility, impaired mobility, brain atrophy, AUTS2 syndrome, unknown)	33 (35%)

### Qualitative Results and Emerging Themes

The qualitative interviews revealed several themes related to HL among caregivers.

#### Understanding and Awareness of Diagnosis (Functional HL)

Among Arabic-speaking caregivers, many expressed difficulties in comprehending their child's diagnosis, often relying on translators or external resources. One participant shared, “*I had a translator to help me understand. I totally don't understand the diagnosis…* *I am not fully aware of my son's case; if anybody asks a question, I don’t know how to answer.”* Conversely, English-speaking caregivers demonstrated a better grasp of their child's condition, often attributing their knowledge to online support groups. As one participant stated, “*Yes, I do. I understand the diagnosis very well. It's a very, very rare disease. There are hardly 150 patients in the world with this disease. So, as much information as there is, we understand it.”*

#### Seeking External Resources for Clarity (Critical HL)

Arabic-speaking caregivers frequently turned to online sources such as Google to understand medical terminology and possible outcomes, indicating a disconnect between their perceived understanding and their reported HL levels. One Arabic-speaking caregiver explained, “*So, I just tried to check on Google with my husband to try and understand what exactly it meant. In terms of images, how does it look? What are the outcomes of it?”* In contrast, English-speaking caregivers appeared more confident in their understanding and relied less on external resources, aligning with survey results. One English-speaking caregiver noted, “*I didn’t need to check online; my doctor explained everything to me very well.”*

#### Reliance on Physician Guidance (Empowerment HL)

Both Arabic- and English-speaking caregivers emphasized following healthcare professionals’ recommendations as their primary approach to managing their child's condition. One Arabic-speaking caregiver stated, “*For now, I don’t have a specific answer to what's best for my child because all I know is that we’re following the doctors’ and physiotherapists’ instructions.”* Similarly, an English-speaking caregiver remarked, “*I asked the doctor twice, and they explained everything. My doctor was amazing. He helped me so much and explained each and everything briefly.”* This suggests a strong trust in medical providers but a potential lack of active decision making in managing their child's health.

#### Active Communication with Physicians (Communicative HL)

Participants across both language groups expressed confidence in their ability to communicate with physicians, with many stating they would ask for repeated explanations until they fully understood the information provided. One Arabic-speaking caregiver shared, “*Yes, for everything I could understand, they explained to me again and again. If I did not understand, I could ask them because I wanted to know what they were going to do with my child.”* Similarly, an English-speaking caregiver described their approach, “*I ask a lot of questions. I don’t go back home with lingering doubts. No matter how many times they have to explain, I ask because it's about my child's health. It's not about me looking smart in front of them; it's about understanding fully so I can do what's right for my child.”*

These themes illustrate how caregivers navigate HL challenges and how their experiences align with or diverge from the quantitative survey results. The insights gained inform the subsequent integration of quantitative and qualitative findings.

### Integration of Quantitative and Qualitative Results of HL Domains

Table 3 presents a comprehensive analysis of the joint display of quantitative and qualitative data, categorizing significant findings according to the domains of the AAHL scale: functional HL, critical HL, empowerment, and communicative HL. In the Functional HL domain, the Arabic sample displayed discordance with a mean score of 7.1 ± 1.4 (out of 9), revealing challenges in understanding their child's diagnosis. Conversely, the English sample exhibited expansion with a mean score of 7.5 ± 1.5, indicating a nuanced comprehension of health-related information. Transitioning to the critical HL domain, the Arabic sample showed discordance (9.0 ± 1.7 out of 12), mentioning external resources like Google, contrary to survey responses. In contrast, the English sample demonstrated expansion (10.0 ± 1.4), aligning with AAHL scores, as no outside resources were reported. In the Empowerment domain, both Arabic and English samples expanded, with mean scores of 5.7 ± 0.9 and 5.4 ± 1.0 (out of 7), respectively. Participants stressed the significance of following physicians’ instructions for optimal treatment, indicating a congruence between qualitative and quantitative findings. In the Communicative HL domain, both samples expanded, with mean scores of 8.2 ± 1.0 (Arabic) and 8.6 ± 0.8 (English) out of 7. Participants highlighted the importance of clear communication with physicians and expressed a proactive approach in seeking clarifications through questions, aligning with AAHL scores.

**Table 3.** Joint Display of Quantitative and Qualitative Data with Meta-Inferences of HL Domains.

MM domains HL scores (mean ± SD)	Qualitative findings	Quantitative findings
Functional health literacy domain	Arabic sample	Discordance
7.1 ± 1.4 (out of 9)	*e.g.: “I had a translator to help me understand. I totally don't understand the diagnosis… and I am not fully aware about my son case if anybody ask a question I don't know how to answer.”*	In accordance with the AAHLS scores, parents did not fully understand their child's diagnosis and, therefore, did not clarify what they didn't understand
	English sample	Expansion
7.5 ± 1.5 (out of 9)	*e.g.: “Yes, I understand the basics of it because It's something very complicated. … I understand it from the groups that I follow.”* *e.g.,: “… I understand the diagnosis very well. It's a very, very rare disease. There are hardly 150 patients in the world with this disease. … as much as information that's there, we understand that, yeah."*	Responses from participants were congruent with the AAHLS results
Critical health literacy domain	Arabic sample	Discordance
9.0 ± 1.7 (out of 12)	*e.g.: “ I just tried to check on Google with my husband to try and understand what exactly was it meaning…"*	Participants stated they used outside resources, which is incongruent with survey responses
	English sample	Expansion
10.0 ± 1.4 (out of 12)	*No outside resources used*	AAHLS demonstrate that this sample is congruent with scores
Empowerment domain	Arabic sample	Expansion
5.7 ± 0.9 (out of 7)	*e.g.: “The best way to help her improve? I cannot say. For now, I don't have a specific answer to that because all I know is just that we're following instructions from the doctors and the physiotherapist.”*	Participants from both the English and Arabic samples felt that following physicians’ instructions was the best way to help their child with the best possible treatment for the disease
	English sample	Expansion
5.4 ± 1.0 (out of 7)	*e.g.: “I asked the doctor twice. They explained me. … he helped me so much. He explained everything. Each and everything briefly.”*	Participants from both the English and Arabic samples felt that following physicians’ instructions was the best way to help their child with the best possible treatment for the disease
Communicative health literacy domain	Arabic sample	Expansion
8.2 ± 1.0 (out of 9)	*e.g.: “For everything I could understand, they could explain to me again and again. If I did not understand, I could ask them because you want to know what they're going to do with my child.”*	In accordance with AAHL scores, participants felt the physician explained things so that they understood the intended message
	English sample	Expansion
8.6 ± 0.8 (out of 9)	*e.g.,: “Usually, I ask a lot of questions. I don't go back home with some sort of a lingering question in mind. …. It's not about whether I look smart in front of them or not. It's about whether I understood it right, so I could do what's right for my child. … generally, [I] do not leave the place if I'm unsure.”*	In accordance with AAHL scores, participants felt the physician explained things so that they understood the intended message

AAHL = All Aspects of Health Literacy Scale.

## Discussion

This mixed-method study, conducted in a pediatric rehabilitation specialty clinic in Qatar, is among the first to assess HL levels among Arabic- and English-speaking parents. Overall, the findings from the AAHL survey and qualitative interviews indicated high overall HL levels, though notable differences exist across functional, communicative, and critical HL domains. These results highlight the need for tailored HL interventions, particularly for non-English-speaking caregivers, to ensure better access to health information and improved pediatric healthcare outcomes (Nair et al., 2022).

The study sample included a diverse caregiver population with varying educational backgrounds and socioeconomic statuses. The predominance of caregivers aged 30 to 39 years suggests that many parents are managing both career and child-rearing responsibilities, making time a limiting factor in health information-seeking behaviors (Leach et al., 2023). The income distribution highlights economic disparities, suggesting that HL interventions should be adaptable to different financial backgrounds (Hasannejadasl et al., 2022; Johnson et al., 2024). Additionally, the prevalence of cerebral palsy and genetic disorders among the children in this study emphasizes the need for specialized, condition-specific HL support, particularly for parents navigating complex treatment plans (Gill et al., 2021).

A key finding is the difference in functional HL between Arabic- and English-speaking caregivers. Arabic-speaking parents struggled to comprehend medical diagnoses, often relying on translators or unverified online sources for additional information. This aligns with research indicating that linguistic and cultural barriers hinder HL and medical comprehension (Allen et al., 2020; Johnson et al., 2022; Peprah et al., 2024). Limited access to reliable, language-appropriate healthcare materials further exacerbates these challenges, making it difficult for non-English-speaking caregivers to fully understand their child's condition and treatment.

The observed discrepancy between Arabic-speaking caregivers’ self-reported confidence and their actual struggles with functional and critical HL reveals an important tension. This discordance may stem from overestimation of HL or social desirability bias, reflecting a perceived need to demonstrate understanding in a clinical environment (Canady & Larzo, 2023). Such findings call for closer examination of parent caregivers perceived versus measured competencies and reinforce the need for validated assessment tools. Furthermore, English-speaking caregivers’ ability to communicate fluently and their apparent familiarity with medical terminology and concepts likely contributed to their increased agency in healthcare interactions (Pandey et al., 2021). Their experiences underscore how linguistic alignment and cultural congruence with health providers can enhance comprehension, advocacy, and engagement in decision-making processes (Johnson et al., 2022; Pandey et al., 2021).

In contrast, English-speaking parents demonstrated greater confidence in navigating health information, benefiting from direct communication with healthcare providers without language-related obstacles. Their ability to ask questions, seek clarifications, and actively engage in discussions significantly improved their understanding of their child's medical needs. Additionally, their unrestricted access to English-language health resources further facilitated their ability to verify, interpret, and apply health information effectively, reinforcing their HL advantage (Allen et al., 2020; Johnson et al., 2022).

Similarly, critical HL varied between groups. Arabic-speaking parents relied more on external sources, such as Google, for medical information, demonstrating a lack of access to provider-recommended health information This finding is consistent with literature indicating that low HL levels increase reliance on unverified digital sources, which may impact caregivers’ decision-making abilities (Hasannejadasl et al., 2022; Lopes & Ramos, 2020). Conversely, English-speaking parents placed more trust in physician-provided materials, reflecting a higher level of confidence in healthcare communication (Leach et al., 2023).

Both groups demonstrated strong empowerment HL, emphasizing trust in medical professionals and adherence to physician recommendations. Moreover, relatively few caregivers actively engaged in medical decision making, underscoring the need for HL interventions that encourage greater parental involvement (de Buhr & Tannen, 2020; Leach et al., 2023). The high communicative HL scores suggest that caregivers are proactive in seeking clarification from healthcare providers, which aligns with studies showing that caregiver-provider communication is a key determinant of pediatric health outcomes (Gill et al., 2021; Paynter et al., 2022). However, it is important to critically reflect on the nature of empowerment observed in this study. Participants may have equated empowerment with compliance and trust in healthcare providers rather than active involvement in treatment planning or autonomous decision making (Halvorsen et al., 2020). This pattern suggests a more passive form of empowerment that seems to be rooted in respect to medical authority. To foster genuine empowerment, future interventions should promote shared decision making and caregiver participation, equipping families with the skills to question, challenge, and collaborate meaningfully with the healthcare providers.

To the best of our knowledge, this mixed-method study stands out as a distinctive endeavor in the realm of Arab countries. Unlike the majority of previous studies focusing on HL in healthcare settings, which primarily recruited healthcare providers or adult patients, our research unfolded in a pediatric rehabilitation specialty clinic in Qatar. Here, our aim was to assess HL within a self-selected group of parents. When comparing the findings of this study with previous investigations conducted in neighboring countries to assess HL in healthcare settings, a noteworthy distinction emerged. The observed high degree of HL in our survey diverges from the outcomes reported in the region. These differences may be attributed to variations in survey tools and the composition of patient populations (Murugesu et al., 2022; Nair et al., 2022; Siddiqui, 2017). Comparative studies in the United Arab Emirates (Nair et al., 2022) and Saudi Arabia (Alahmadi, 2023) utilizing different HL assessment tools reported considerably lower levels of adequate HL among surveyed patients. Similarly, a recent Qatar-based study on men with acute coronary syndrome and heart failure found a prevalence of inadequate to marginal HL ranging from 36% to 54% (Elbashir et al., 2023). Another study from Bahrain investigating the adult population's HL, showing variability in HL levels across the region (Asokan et al., 2020).

### Research Implications and Recommendations

In light of the comprehensive insights gained into HL domains among caregivers in a pediatric rehabilitation setting, there is a compelling call to action for the development and implementation of tailored HL interventions. These initiatives, strategically designed to address specific challenges identified in the study, particularly the discordance in functional HL among the Arabic-speaking sample, can play a pivotal role. Through targeted educational programs and materials, caregivers stand to enhance their understanding of their child's diagnosis, fostering a more informed and engaged approach to caregiving (Gill et al., 2021; Nair et al., 2022; Siddiqui, 2017).

The linguistic diversity inherent in the caregiver population underscores the necessity of implementing multilingual and culturally responsive communication strategies (Johnson et al., 2022). It is imperative for healthcare providers to receive training that enables effective communication with caregivers across different languages. This approach ensures that information is not only clear and accessible but also culturally sensitive, thereby contributing to an environment where communicative HL thrives and collaboration in healthcare becomes more seamless. Furthermore, the observed discordance in critical HL, particularly the reliance on external resources among Arabic-speaking parents, underscores the need for targeted interventions promoting critical HL skills. Educational initiatives that guide caregivers toward reliable sources of health information and empower them to make well-informed decisions without solely relying on external resources are essential (Nair et al., 2022).

Additionally, co-production of health education materials through participatory approaches such as caregiver-provider collaboration, focus groups, and community-based workshops should be prioritized (Grindell et al., 2022). Such strategies ensure that HL interventions are culturally appropriate, linguistically appropriate, and aligned with caregivers’ experiences and needs. These efforts could also improve trust and message relevance, particularly among Arabic-speaking caregivers. Community-based participatory research (CBPR) frameworks may offer practical solution in designing these inclusive and culturally responsive HL initiatives (Chanchien Parajón et al., 2021; Chen et al., 2020).

Recognizing the prevalent use of external resources, such as Google, by some caregivers, there exists an opportunity to incorporate technology into HL initiatives. The development of user-friendly and reliable online resources, tailored to the specific needs of caregivers, can bridge potential gaps in critical HL, providing them with accurate information and support (Hasannejadasl et al., 2022). To ensure the sustained effectiveness of implemented interventions and ongoing improvements in HL, conducting continued assessments and longitudinal studies is recommended. Regular evaluations will offer valuable insights into the evolving HL landscape among caregivers, allowing for the refinement of interventions and adaptation of strategies to meet changing needs over time.

Collaboration with community organizations and support groups can significantly enhance the reach and impact of HL initiatives. Engaging caregivers through community-based programs, workshops, and outreach activities fosters a supportive network, facilitating the exchange of experiences and knowledge. Lastly, for a lasting impact, the integration of HL into routine clinical practice is crucial. Healthcare providers should undergo training to recognize and address HL challenges, ensuring that communication strategies remain consistently patient-centered, clear, and tailored to individual needs (Murugesu et al., 2022).

In embracing these recommendations, healthcare institutions can actively contribute to building a more health-literate environment. By empowering caregivers to actively participate in their child's healthcare decisions, these initiatives hold the potential to ultimately improve pediatric healthcare outcomes.

### Study Limitations

While this study provides valuable insights into HL among pediatric caregivers in a Qatari rehabilitation clinic, several limitations should be considered. First, the self-selected sample may have introduced response bias, as parents more confident in their health knowledge were likely to participate, potentially inflating HL levels. Second, the study was limited to a single setting, reducing the generalizability of findings. Additionally, although language group and caregiver roles were recorded, other demographic variables such as migration status, length of residency in Qatar, and detailed socioeconomic indicators were not collected, limiting the transferability of qualitative findings. Future studies should consider capturing such data to explore the influence of structural and social determinants on HL.

While descriptive statistics provided valuable insights on HL among this sample population, the lack of inferential analysis restricted exploration of associations between HL and sociodemographic variables like education, income, or professional background. Future research should aim to identify significant predictors of HL using inferential statistics. Moreover, the study's sample leaned toward higher socioeconomic groups, which may not reflect the broader diversity of Qatar's population, therefore, expanding participant diversity in future studies is recommended.

Finally, the reliance on self-reported survey responses raises concerns about how well HL levels translate into actual healthcare behaviors and outcomes. Given the cross-sectional design, future longitudinal studies are needed to track HL changes over time and assess the long-term impact of HL interventions.

## Conclusion

This mixed-method study provides a comprehensive exploration of HL among parents in a pediatric rehabilitation specialty clinic in Qatar. The congruence between quantitative data obtained through the AAHL survey and qualitative insights from personal interviews reveals a robust HL environment among both Arabic- and English-speaking parents. The participants demonstrated strength in communicative HL, actively engaging with healthcare providers. Critical HL showed variability, with Arabic-speaking parents seeking external resources, while English-speaking parents did not. The empowerment domain revealed a shared commitment to participating in healthcare decisions. The study's comprehensive exploration of HL domains, coupled with considerations for demographic diversity, highlights the need for tailored interventions to enhance communication and empower caregivers in pediatric healthcare settings.

## Supplemental Material

sj-docx-1-son-10.1177_23779608251362293 - Supplemental material for Exploring Health Literacy Among Parents of Children Who Attended the Pediatric Rehabilitation Clinics in Qatar: A Convergent Parallel Research Design Supplemental material, sj-docx-1-son-10.1177_23779608251362293 for Exploring Health Literacy Among Parents of Children Who Attended the Pediatric Rehabilitation Clinics in Qatar: A Convergent Parallel Research Design by Jessie Johnson, Fadi Khraim, Carolyn Wolsey, Rami A. Elshatarat, Lisa Thornton, Dina Schnurman, Mohammed AlDalaykeh and Amal Al-Farsi in SAGE Open Nursing

sj-docx-2-son-10.1177_23779608251362293 - Supplemental material for Exploring Health Literacy Among Parents of Children Who Attended the Pediatric Rehabilitation Clinics in Qatar: A Convergent Parallel Research Design Supplemental material, sj-docx-2-son-10.1177_23779608251362293 for Exploring Health Literacy Among Parents of Children Who Attended the Pediatric Rehabilitation Clinics in Qatar: A Convergent Parallel Research Design by Jessie Johnson, Fadi Khraim, Carolyn Wolsey, Rami A. Elshatarat, Lisa Thornton, Dina Schnurman, Mohammed AlDalaykeh and Amal Al-Farsi in SAGE Open Nursing
