# Supplementary material for: Exploring Health Literacy Among Parents of Children Who Attended the Pediatric Rehabilitation Clinics in Qatar: A Convergent Parallel Research Design
Source: SAGE Open Nurs. 2025 Jul 23;11:23779608251362293. doi: 10.1177/23779608251362293 (PMC12301597; doi:10.1177/23779608251362293)
Supplement: sj-docx-1-son-10.1177_23779608251362293 - Supplemental material for Exploring Health Literacy Among Parents of Children Who Attended the Pediatric Rehabilitation Clinics in Qatar: A Convergent Parallel Research Design [file sj-docx-1-son-10.1177_23779608251362293.docx]

- 1. Do you know the name of your child's diagnosis?
  2. Do you feel that you understand your child's diagnosis?
  3. What challenges, if any, have you faced in understanding medical information about your child’s care?
  4. How comfortable do you feel asking questions or seeking clarifications from your child’s doctors or therapists?
  5. Do you use outside resources like Google to help you understand your child's condition or the recommendations that are made by your doctor or therapist?
  6. How do you decide whether the health information you receive (from doctors, online, or other sources) is reliable?
  7. Do you understand the best ways to help your child improve?
  8. Is there any part of the instructions that you are unsure of or uncomfortable doing? What do you do if you are unsure or uncomfortable with an instruction or recommendation?
