# Supplementary material for: Exploring Health Literacy Among Parents of Children Who Attended the Pediatric Rehabilitation Clinics in Qatar: A Convergent Parallel Research Design
Source: SAGE Open Nurs. 2025 Jul 23;11:23779608251362293. doi: 10.1177/23779608251362293 (PMC12301597; doi:10.1177/23779608251362293)
Supplement: sj-docx-2-son-10.1177_23779608251362293 - Supplemental material for Exploring Health Literacy Among Parents of Children Who Attended the Pediatric Rehabilitation Clinics in Qatar: A Convergent Parallel Research Design [file sj-docx-2-son-10.1177_23779608251362293.docx]

**Domain 1: Research Team and Reflexivity**

| **No.** | **Item** | **Description** | **Response** |
| --- | --- | --- | --- |
| **1** | Interviewer/facilitator | Who conducted the interviews? | The interviews were conducted by two researchers with qualitative research expertise. |
| **2** | Credentials | What were the interviewer’s credentials? | Both interviewers held graduate degrees in healthcare and qualitative research. |
| **3** | Occupation | What was their role in the study? | The interviewers were researchers involved in data collection and analysis. |
| **4** | Gender | What was the interviewer's gender? | Both interviewers were females. |
| **5** | Experience & training | What was the interviewer’s experience/training in qualitative research? | Both researchers had prior qualitative research experience and received training in interview techniques. |
| **6** | Relationship with participants | Was a relationship established prior to study commencement? | No prior relationship existed between the interviewers and participants. |
| **7** | Participant knowledge of interviewer | What did participants know about the interviewer? | Participants knew that the interviewer was a researcher studying parental HL. |
| **8** | Interviewer characteristics | What characteristics were relevant to the study? | Interviewers remained neutral and objective during data collection. |

**Domain 2: Study Design**

| **No.** | **Item** | **Description** | **Response** |
| --- | --- | --- | --- |
| **9** | Methodological orientation | What qualitative approach was used? | A convergent parallel mixed-methods design was applied. |
| **10** | Sampling | How were participants selected? | Convenience sampling was used, targeting parents of children attending a pediatric rehabilitation clinic. |
| **11** | Method of approach | How were participants recruited? | Recruitment occurred via email invitations and direct invitations at clinic visits. |
| **12** | Sample size | How many participants were included? | 16 parents (9 English-speaking, 7 Arabic-speaking). |
| **13** | Non-participation | How many refused to participate? | No participants refused to participate. |
| **14** | Setting | Where was the study conducted? | In a pediatric rehabilitation clinic in Doha, Qatar. |
| **15** | Presence of non-participants | Were others present during interviews? | No, interviews were conducted privately. |
| **16** | Description of sample | Key demographic details | Participants were parents of children with disabilities (e.g., cerebral palsy, genetic disorders). |
| **17** | Interview guide | Was an interview guide used? | Yes, a semi-structured interview guide was used. |
| **18** | Repeat interviews | Were repeat interviews conducted? | No repeat interviews were conducted. |
| **19** | Audio/visual recording | Were interviews recorded? | Yes, all interviews were audio-recorded with consent. |
| **20** | Field notes | Were field notes made? | Yes, field notes were taken during interviews. |
| **21** | Duration | How long were the interviews? | 20–45 minutes per interview. |
| **22** | Data saturation | Was data saturation discussed? | Yes, data saturation was achieved after 16 interviews. |
| **23** | Transcripts returned | Were transcripts returned for participant validation? | No, transcripts were not returned to participants. |

**Domain 3: Data Analysis & Reporting**

| **No.** | **Item** | **Description** | **Response** |
| --- | --- | --- | --- |
| **24** | Data analysis approach | What method was used? | Thematic analysis following Gale et al.’s framework (2013). |
| **25** | Coding process | Who coded the data? | Two independent researchers coded the data. |
| **26** | Software | What software was used? | Manual analysis (no software used). |
| **27** | Participant checking | Did participants provide feedback on findings? | No, participant checking was not conducted. |
| **28** | Quotations | Were participant quotes used? | Yes, direct quotes were included in the results. |
| **29** | Data presentation | How were findings presented? | Findings were presented in narrative and tabular formats. |
| **30** | Clarity of themes | Were themes clearly identified? | Yes, themes were clearly defined and linked to research questions. |
| **31** | Consistency | Was consistency maintained in theme analysis? | Yes, coding was reviewed independently for consistency. |
| **32** | Reflexivity | Were biases discussed? | Yes, multiple coders were used to minimize bias. |
